# Spontaneous intramural small-bowel hematoma secondary to coagulopathy in patients receiving vitamin K antagonists: A case series report

**DOI:** 10.1097/MD.0000000000048527

**Published:** 2026-05-01

**Authors:** Khac Thao Thai, Trong Hoe Nguyen, Thanh Son Le, Doanh Hieu Tran

**Affiliations:** a Department of Gastrointestinal Surgery, Military Hospital 103, Hanoi, Vietnam; b Department of Hepatobiliary and Pancreatic Surgery, Military Hospital 103, Hanoi, Vietnam.

**Keywords:** coagulopathy, Spontaneous intramural small-bowel hematoma, vitamin K antagonists

## Abstract

**Rationale:**

Spontaneous intramural small-bowel hematoma (SISBH) is a rare but potentially life-threatening complication of anticoagulant therapy and severe coagulopathy. Because it may mimic acute abdomen or intestinal obstruction, delayed recognition can lead to unnecessary surgery. This study aimed to describe the clinical characteristics, imaging findings, and outcomes of patients with SISBH secondary to vitamin K antagonist-related coagulopathy.

**Patient concerns:**

We retrospectively analyzed 11 consecutive patients with SISBH secondary to vitamin K antagonist-related coagulopathy, including available follow-up data up to 3 months after treatment. The median age was 65 years, and 72.3% were male. All patients presented with acute abdominal pain. Abdominal distension occurred in 63.6%, vomiting in 45.5%, and gastrointestinal bleeding in 9.1%.

**Diagnoses:**

All patients had markedly abnormal coagulation profiles, with a median international normalized ratio (INR) of 8.5 and a median prothrombin activity of 8%. Contrast-enhanced computed tomography revealed circumferential bowel wall thickening with increased intramural attenuation and luminal narrowing in all cases. Partial intestinal obstruction was identified in 54.5%.

**Interventions:**

Ten patients (90.9%) were treated conservatively with discontinuation of anticoagulants, intravenous vitamin K, and fresh frozen plasma. One patient underwent diagnostic laparoscopy without bowel resection.

**Outcomes:**

Coagulation parameters improved significantly after treatment (INR, *P* = .003). The median hospital stay was 6 days. No in-hospital mortality occurred. During the 3-month follow-up period, 1 patient experienced recurrence.

**Lessons:**

Markedly elevated INR in patients with acute abdominal pain should raise suspicion for SISBH. Early computed tomography evaluation and timely correction of coagulopathy may facilitate conservative management and reduce the need for surgical exploration in selected cases.

## 1. Introduction

Anticoagulant therapy is widely used for the prevention and treatment of thromboembolic disorders, particularly in patients with atrial fibrillation, mechanical heart valves, or a history of venous thromboembolism. Although effective in reducing thrombotic events, anticoagulation therapy is associated with an increased risk of bleeding complications, which remain the most clinically significant adverse events. The reported incidence of bleeding among patients receiving anticoagulants ranges from 5% to 48%, with gastrointestinal bleeding occurring in approximately 2% to 4% of cases.^[1,2]^

Spontaneous intramural small-bowel hematoma (SISBH) is an uncommon manifestation of anticoagulant-related bleeding and has been estimated to occur in approximately 1 in 2500 patients receiving anticoagulation therapy.^[3]^ Among available anticoagulants, vitamin K antagonists such as warfarin and acenocoumarol remain leading causes of major bleeding and gastrointestinal hemorrhage in several comparative studies, whereas heparin and direct oral anticoagulants may demonstrate similar or lower bleeding profiles in certain clinical settings.^[4,5]^

The pathophysiology of SISBH involves bleeding into the submucosal or muscular layers of the intestinal wall, resulting in bowel wall thickening and luminal narrowing. Clinically, patients may present with abdominal pain, nausea, vomiting, abdominal distension, or gastrointestinal bleeding. Because of its rarity and nonspecific clinical presentation, SISBH is frequently misdiagnosed or diagnosed late. Early recognition and timely CT imaging are essential for accurate diagnosis and appropriate management.^[6,7]^

Historically, SISBH was first described in 1838 by McLauchlan in a case of duodenal obstruction identified at autopsy. Radiologic characterization emerged more than a century later. To date, published data remain limited and are predominantly case reports or small case series. Abbas et al reported 13 cases in Scotland between 1983 and 2000, while subsequent series from Turkey and South Korea described 15 and 37 cases, respectively, most of whom were receiving anticoagulant or antiplatelet therapy.^[8-10]^ A recent MEDLINE-based review identified 103 reported cases over a 30-year period, underscoring the rarity of this condition.^[10]^

Therefore, we conducted a retrospective case series of 11 patients with SISBH secondary to coagulopathy to describe their clinical presentation, imaging characteristics, and treatment outcomes.

## 2. Materials and methods

### 2.1. Case design and patients

This study was designed as a single-center retrospective case series. Patients were consecutively admitted between April 21, 2023, and September 5, 2025, at the Department of Gastrointestinal Surgery, Military Hospital 103. All patients were followed for 3 months after treatment, and follow-up for the last enrolled patient was completed in January 2026. All patients presented with acute abdominal symptoms and laboratory evidence of coagulopathy while receiving vitamin K antagonist therapy, and were screened through electronic medical records. Spontaneous intramural small-bowel hematoma (SISBH) was diagnosed primarily based on contrast-enhanced abdominal computed tomography (CT) findings.

Patients were included if they met the following criteria: current or prior use of vitamin K antagonists (e.g., warfarin or acenocoumarol); presentation with acute abdominal pain, with or without nausea, vomiting, or gastrointestinal bleeding; contrast-enhanced abdominal CT findings consistent with intramural intestinal hematoma, including circumferential bowel wall thickening, increased intramural attenuation, and luminal narrowing causing partial or complete obstruction; and international normalized ratio (INR) exceeding the recommended therapeutic range at admission. Patients were excluded if the intramural hematoma was related to trauma, intestinal tumors, inflammatory bowel disease, mesenteric ischemia, disseminated intravascular coagulation, severe liver disease, or if medical records were incomplete.

This retrospective study was approved by the Ethics Committee of Military Hospital 103. Written informed consent for publication of anonymized clinical information and images was obtained in accordance with institutional requirements.

### 2.2. Data collection and analysis

Retrospective clinical data were collected from inpatient and electronic medical records. Demographic characteristics included age, sex, body mass index, comorbidities, and indications for vitamin K antagonist therapy.

Clinical presentation data included abdominal pain, vomiting, abdominal distension, signs of peritoneal irritation, and evidence of gastrointestinal bleeding. Laboratory parameters comprised complete blood count (white blood cell count, neutrophil count, red blood cell count, hemoglobin, platelet count), renal function tests (urea, creatinine), and coagulation profiles before and after treatment (prothrombin activity [PT], INR, activated partial thromboplastin time).

Radiologic findings were evaluated based on contrast-enhanced abdominal CT scans, including the location of bowel involvement (duodenum, jejunum, ileum) and the number of affected segments.

Treatment strategies were categorized as conservative management or surgical intervention. Clinical outcomes included recovery status, complications, length of hospital stay, and in-hospital mortality.

Statistical analysis was performed using SPSS version 22.0 (IBM Corp., Armonk). Continuous variables were presented as median and interquartile range (IQR: Q1–Q3), and categorical variables as percentages. Comparisons between pre- and posttreatment parameters were performed using the Wilcoxon signed-rank test. A *P* value < .05 was considered statistically significant.

## 3. Results

### 3.1. Patient characteristics

A total of 11 consecutive patients were included in this study. The median age was 65 years (interquartile range [IQR], 55–74 years). Eight patients (72.3%) were male and 3 (27.7%) were female. Seven patients (63.6%) had underlying comorbidities, most commonly hypertension. Indications for vitamin K antagonist therapy included mechanical heart valve replacement, atrial fibrillation, coronary artery stenting, previous arterial thrombosis, heart failure, and dilated cardiomyopathy.

### 3.2. Clinical presentation

All patients (100%) presented with acute abdominal pain at admission. Abdominal distension was observed in 7 patients (63.6%), vomiting in 5 (45.5%), and abdominal wall guarding in 3 (27.3%). Gastrointestinal bleeding occurred in 1 patient (9.1%). Detailed individual clinical presentations are summarized in Table 2.

**Table 2:** Individual patient characteristics, initial presentation, treatment, and short-term outcomes.

Patient	Age	Sex	Initial presentation	Pretreatment INR	Management	Outcomes			3-mo follow-up
						Posttreatment INR	Hospital stay (d)	Discharge outcome	
1	65	Female	Abdominal pain	20.1	Conservatively treatment	1.2	5	Improved, discharged	No recurrence
2	55	Male	Abdominal pain, vomiting	6.7	Conservatively treatment	1.6	7	Improved, discharged	No recurrence
3	76	Male	Abdominal pain	13.0	Conservatively treatment	1.4	6	Improved, discharged	No recurrence
4	79	Male	Abdominal pain, abdominal distension, vomiting	2.2	Conservatively treatment	1.3	5	Improved, discharged	No recurrence
5	74	Male	Abdominal pain	7.1	Conservatively treatment	2.1	5	Improved, discharged	No recurrence
6	71	Male	Abdominal pain, abdominal distension, vomiting, abdominal wall guarding	3.5	Conservatively treatment	1.3	5	Improved, discharged	No recurrence
7	53	Female	Abdominal pain, abdominal distension, vomiting	20.4	Conservatively treatment	1.2	6	Improved, discharged	No recurrence
8	63	Male	Abdominal pain, gastrointestinal bleeding, abdominal distension	8.5	Conservatively treatment	1.0	9	Improved, discharged	No recurrence
9	67	Male	Abdominal pain, abdominal distension, vomiting, abdominal wall guarding	20.3	Laparoscopic exploration and no bowel resection	1.0	8	Improved, discharged	No recurrence
10	58	Male	Abdominal pain, abdominal distension, abdominal wall guarding	25.0	Conservatively treatment	1.3	7	Improved, discharged	Recurrence at 3 mo; readmitted for medical treatment
11	51	Female	Abdominal pain, abdominal distension	4.1	Conservatively treatment	1.3	7	Improved, discharged	No recurrence

APTT *=* activated partial thromboplastin time (s), INR *=* international normalized ratio, IQR *=* interquartile range, PT *=* prothrombin activity.

### 3.3. Laboratory findings

On admission, laboratory evaluation revealed a median white blood cell count of 11.3 G/L (IQR, 8.8–14.8) and a median neutrophil count of 8.8 G/L (IQR, 7.7–13.6). The median red blood cell count was 3.9 T/L (IQR, 3.3–4.4), and the median hemoglobin level was 116 g/L (IQR, 106–131). The median platelet count was 251 G/L (IQR, 165–281).

Renal function tests showed a median urea level of 10.3 mmol/L (IQR, 7.9–15.7) and a median creatinine level of 114.8 µmol/L (IQR, 90.7–164.4).

Coagulation parameters were markedly abnormal at presentation. The median PT was 8% (IQR, 3–11), the median international normalized ratio (INR) was 8.5 (IQR, 4.1–25), and the median activated partial thromboplastin time was 66.1 seconds (IQR, 60.5–109). Changes in coagulation parameters before and after treatment are presented in Table 1.

**Table 1:** Changes in coagulation parameters before and after treatment.

Variable	Before treatment, median (IQR)	After treatment, median (IQR)	*P* value; effect size and median paired difference (95% CI)
PT (%)	8.0 (3–11)	69 (66–80)	*P* value = .003; effect size (*r*) = 0.88; median paired difference = 61 (45–77)
INR	8.5 (4.1–25)	1.3 (1.2–1.4)	*P* value = 0.003; effect size (*r*) = 0.88; median paired difference = 5.1 (2.8–19.2)
APTT (s)	66.1 (60.5–109)	31.6 (29.4–35.6)	*P* value = 0.005; effect size (*r*) = 0.84; median paired difference = 35.3 (23.5–79.6)

*P* values were calculated using the Wilcoxon signed-rank test. Effect size is reported as *r*. The median paired difference and its 95% confidence interval are provided as descriptive interval estimates.

APTT = activated partial thromboplastin time, CI = confidence interval, IQR = interquartile range, INR = international normalized ratio, PT = prothrombin activity.

### 3.4. Computed tomography findings

In this study, the jejunum was the most frequently involved segment (7 patients, 63.6%), followed by the ileum (4 patients, 36.4%). All patients demonstrated characteristic CT features of intramural small-bowel hematoma, including circumferential bowel wall thickening, increased intramural attenuation, and luminal narrowing.

**Figure 1.**
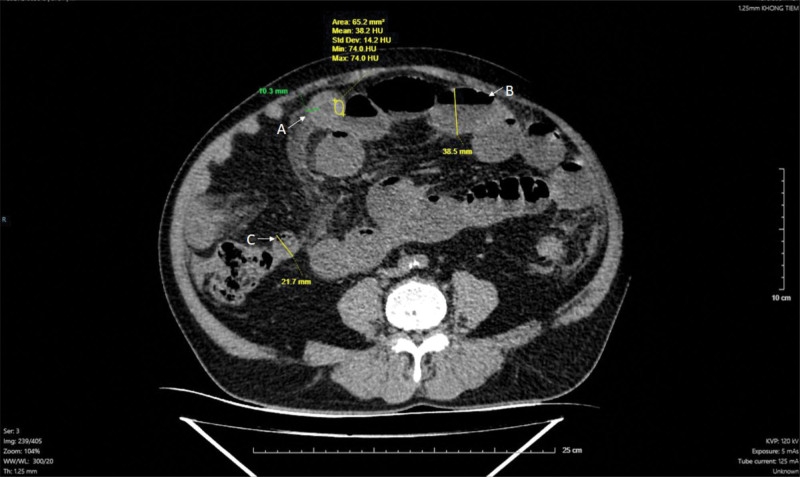
Non-contrast abdominal CT showing intramural small-bowel hematoma. (A) Segmental circumferential bowel wall thickening (approximately 10–12 mm) with increased intramural attenuation (mean ~38 HU, with focal values up to 70 HU), consistent with intramural hematoma. The lesion causes luminal narrowing and demonstrates a characteristic “target sign” appearance. (B) Proximal bowel dilatation with air–fluid levels upstream of the narrowed segment. (C) Collapsed distal bowel segment beyond the site of luminal narrowing. CT = computed tomography.

**Figure 2.**
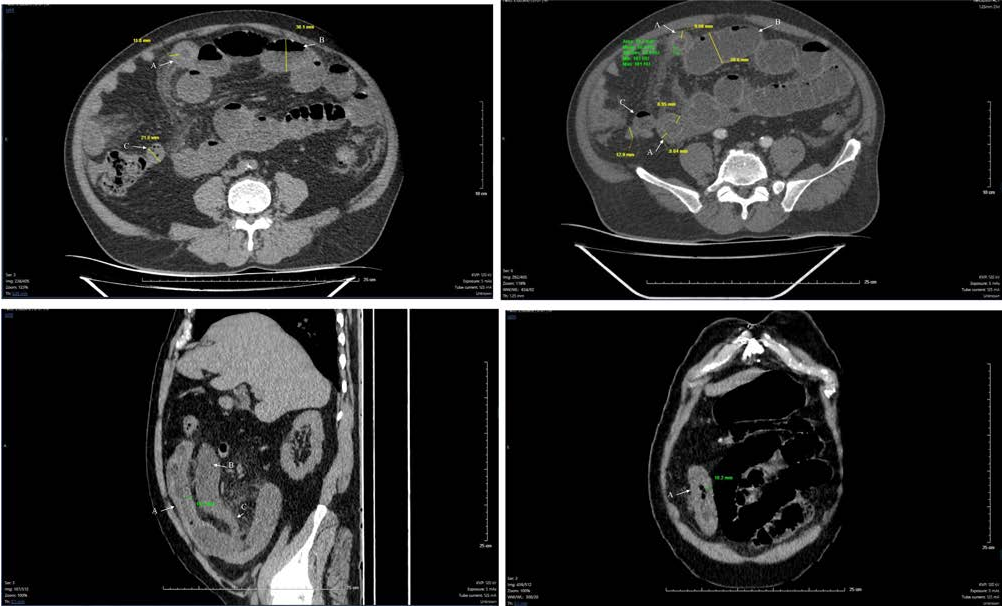
Multiplanar reconstructions. Non-contrast abdominal CT with axial multiplanar reconstruction (MPR). Segmental circumferential small-bowel wall thickening (A) with associated luminal narrowing is observed, accompanied by proximal bowel dilatation (B) and distal bowel collapse (C). Axial contrast-enhanced abdominal CT in the arterial phase (slice thickness 1.25 mm) with quantitative attenuation measurements (HU). The characteristic “target sign” (A) is clearly demonstrated, reflecting layered attenuation of the bowel wall. Quantitative attenuation measurements confirm increased intramural density. Non-contrast abdominal CT with coronal multiplanar reconstruction (MPR). The longitudinal extent of the affected bowel segment (A) is clearly delineated, with associated proximal dilatation (B) and distal collapse (C). Non-contrast abdominal CT with sagittal multiplanar reconstruction (MPR). Segmental bowel wall thickening (A) is demonstrated, clarifying the spatial orientation of the lesion along the bowel axis. CT = computed tomography, MPR = multiplanar reconstruction.

On non-contrast CT (slice thickness 1.25 mm), the affected bowel wall thickness ranged from approximately 10 to 12 mm, with increased attenuation values (mean approximately 38 Hounsfield units, with focal values up to 70 HU), consistent with intramural hematoma (Fig. 1). On contrast-enhanced CT, a characteristic “target sign” was observed, reflecting layered attenuation within the bowel wall, and was most clearly visualized in the arterial phase (Fig. 2).

Multiplanar reconstructions further delineated the segmental involvement and longitudinal extent of the lesion, particularly on axial, coronal, and sagittal views (Fig. 2). Indirect CT findings included features of partial intestinal obstruction, identified in 6 patients (54.5%), characterized by proximal bowel dilatation and distal bowel collapse, as well as intraperitoneal fluid in 8 patients (72.7%; Fig. 1).

### 3.5. Treatment and outcomes

Ten patients (90.9%) were successfully managed conservatively. Treatment included discontinuation of vitamin K antagonists, intravenous vitamin K administration, fresh frozen plasma (FFP) transfusion, bowel rest, and supportive care. One patient (9.1%) underwent laparoscopic exploration because the initial clinical presentation was suggestive of peritonitis, warranting surgical intervention. In this setting, laparoscopy served both as a diagnostic modality to determine the cause of the acute abdomen and as a potential therapeutic approach if a surgically treatable lesion had been identified. Early exploration therefore enabled prompt diagnosis and timely management. Intraoperatively, spontaneous intramural small-bowel hematoma related to coagulopathy was confirmed, with no evidence of bowel perforation, ischemia, or necrosis.

Following treatment, coagulation parameters improved significantly. The median PT increased from 8% to 69% (IQR, 66–80; *P* = .003), while the median INR decreased from 8.5 to 1.3 (IQR, 1.2–1.4; *P* = .003). The median activated partial thromboplastin time decreased from 66.1 seconds to 31.6 seconds (IQR, 29.4–35.6; *P* = .005), based on Wilcoxon signed-rank testing (Table 1).

The median time to resume oral intake was 3 days (IQR, 2–4), the median time to INR normalization was 4 days (IQR, 3–5), and the median length of hospital stay was 6 days (IQR, 5–7). No treatment-related complications or in-hospital mortality were observed. In the patient who underwent laparoscopy, no bowel resection was required, and the postoperative course was uneventful. The patient was discharged after 8 days.

In our practice, oral anticoagulation was resumed after clinical stabilization, resolution of abdominal pain and bleeding symptoms, and normalization or near-normalization of INR, with dose adjustment according to coagulation parameters and thromboembolic risk.

During the 3-month follow-up period, 1 patient developed recurrent SISBH and was readmitted for medical treatment. Recurrence occurred approximately 3 months after the initial episode (October 8, 2025–January 4, 2026), with an INR of 4 at readmission. After clinical stabilization, vitamin K antagonist therapy was continued with dose adjustment based on coagulation parameters. Detailed patient-level data regarding heparin bridging or switching to direct oral anticoagulants after recovery are summarized in Table 2.

A summary of the overall clinical timeline from presentation to 3-month follow-up is provided in Table 3.

**Table 3:** Timeline of clinical presentation, diagnosis, management, and 3-month follow-up.

Time point	Clinical events and assessments
Admission	Patients presented with acute abdominal pain, with or without abdominal distension, vomiting, abdominal wall guarding, or gastrointestinal bleeding
Initial evaluation	Clinical examination, coagulation testing, complete blood count, renal function tests, and contrast-enhanced abdominal computed tomography were performed
Diagnosis	Spontaneous intramural small-bowel hematoma was diagnosed based on vitamin K antagonist exposure, coagulation abnormalities, and characteristic computed tomography findings
Initial treatment	Vitamin K antagonists were discontinued. Conservative treatment consisted of intravenous vitamin K, fresh frozen plasma, bowel rest, and supportive care
Surgical assessment	One patient underwent diagnostic laparoscopy because the initial presentation was suggestive of peritonitis. No bowel resection was required
Early clinical response	The median time to resume oral intake was 3 days (IQR, 2–4), and the median time to INR normalization was 4 days (IQR, 3–5). Coagulation parameters improved significantly after treatment
Discharge	The median length of hospital stay was 6 days (IQR, 5–7). No treatment-related complications or in-hospital mortality were observed
Follow-up (3 mo)	During follow-up, 1 patient developed recurrent SISBH approximately 3 months after the initial episode and was readmitted for medical treatment. The remaining patients had no documented recurrence during the follow-up period

INR *=* international normalized ratio, IQR *=* interquartile range, SISBH *=* spontaneous intramural small-bowel hematoma.

## 4. Discussion

We reported 11 cases of spontaneous intramural small-bowel hematoma (SISBH) related to coagulopathy associated with vitamin K antagonist use. The most common site of hematoma was the jejunum (63.6%), followed by the ileum (36.4%), and no cases involved the duodenum. Abbas et al also found that the jejunum was the most frequently affected site (69%), with lower rates in the ileum and duodenum at 38% and 23%, respectively.^[8]^ In contrast, Kang et al reported that the ileum was the most common site (45.9%), followed by the jejunum (43.2%) and the duodenum (10.8%).^[10]^

The predominant clinical manifestation of SISBH is acute abdominal pain, which occurs in most cases and may be diffuse or localized at the site of obstruction. Symptoms of bowel obstruction or sub-obstruction such as vomiting, abdominal distension, and constipation may also occur. Signs of peritoneal irritation are observed in cases complicated by necrosis, perforation, or intraperitoneal hemorrhage. Gastrointestinal bleeding, most commonly presenting as melena, has also been reported.^[11]^ In our study, all patients presented with abdominal pain at admission. Abdominal distension, vomiting, and abdominal wall guarding were less frequent, occurring in 63.6%, 45.5%, and 27.3% of patients, respectively. Gastrointestinal bleeding was observed in only 1 patient (9.1%). Altintoprak et al reported similar findings, with abdominal pain being the most common symptom (100%), followed by vomiting (53.3%), weakness (40%), and anorexia (26.6%).^[9]^ In contrast, Kang et al found that abdominal pain occurred in 64.9% of cases, gastrointestinal bleeding in 37.8%, and nausea and/or vomiting in 35.1%.^[10]^

Computed tomography (CT) is considered the gold standard for the diagnosis of intramural intestinal hematoma. Typical CT findings include circumferential thickening of the small-bowel wall with increased intramural attenuation corresponding to the site of hemorrhage, and luminal narrowing leading to partial or complete intestinal obstruction.^[5]^ CT imaging also helps differentiate SISBH from conditions requiring surgical intervention, such as bowel necrosis or perforation, which may present with decreased or absent mural enhancement and free intraperitoneal air. In our study, all patients demonstrated characteristic CT findings of circumferential bowel wall thickening with increased intramural attenuation and luminal narrowing. Intestinal obstruction was observed in 54.5% of cases.

The primary management of SISBH is conservative therapy, including discontinuation of vitamin K antagonists, intravenous vitamin K administration, and FFP transfusion. Surgical intervention is generally reserved for patients who develop severe complications such as persistent intestinal obstruction, perforation, ischemia, peritonitis, or uncontrolled intra-abdominal bleeding despite adequate medical treatment.^[10]^

Because SISBH may clinically mimic acute abdomen or bowel obstruction, diagnostic uncertainty can lead to surgical exploration. In our series, 10 patients (90.9%) were successfully managed conservatively, whereas 1 patient (9.1%) underwent diagnostic laparoscopy due to suspected peritonitis of unknown origin. No bowel resection was required, and the postoperative course was uneventful. Comparable outcomes were reported by Altintoprak et al, in which 86.6% of patients were treated non-operatively and 13.3% required surgery.^[9]^

The median length of hospital stay in our study was 6 days, shorter than the mean hospital stay of 9.35 days reported by Kang et al.^[10]^ No mortality occurred during hospitalization. In contrast, Altintoprak et al reported a mortality rate of 13.3%,^[9]^ while the MEDLINE-based literature review by Kang et al, which included 103 reported cases of SISBH, found an overall mortality rate of 5.8%, mainly due to sepsis or multi-organ failure.^[10]^ Coagulation parameters improved significantly following treatment, supporting the effectiveness of conservative management in the acute phase.

Resumption of anticoagulation after recovery should be individualized according to bleeding control, INR normalization, and the patient’s underlying thromboembolic risk. In patients at high thromboembolic risk, bridging therapy with short-acting parenteral anticoagulants may be considered once adequate hemostasis has been achieved, because such agents can be rapidly discontinued if rebleeding occurs. In lower-risk patients, anticoagulation may be resumed without bridging after clinical stabilization. In our retrospective series, however, detailed patient-level data on bridging strategies or switching to direct oral anticoagulants during follow-up were not uniformly documented. Vitamin K antagonists remain a classic cause of SISBH; however, the epidemiology of anticoagulant-associated bowel hematoma is evolving as direct oral anticoagulants are increasingly used in routine clinical practice.

Recent reports have described DOAC-associated intramural intestinal hematoma, while contemporary small-bowel case reports continue to highlight this condition in anticoagulated patients and underscore the need for prompt recognition and appropriate management.^[5,12]^ However, contemporary data remain limited, and most published experience still consists of case reports and small retrospective series.^[1,10,13]^ Current guidance on anticoagulant-associated gastrointestinal bleeding emphasizes prompt reversal of coagulopathy, hemodynamic stabilization, and individualized reassessment of thromboembolic risk before restarting anticoagulation.^[14]^ In SISBH, markedly elevated INR remains an important diagnostic clue and may also reflect bleeding severity; however, robust INR-based risk stratification is still limited because the available evidence is derived mainly from small retrospective series and case reports.^[1,9,10]^ In Vietnam, the available literature has focused mainly on the broader clinical characteristics of patients receiving oral anticoagulants who present with bleeding, rather than on SISBH specifically.^[15]^ This regional paucity of data highlights the relevance of the present case series.

## 5. Conclusion

We reported 11 rare cases of spontaneous intramural intestinal hematoma associated with coagulopathy. The diagnosis was based on acute abdominal pain, coagulation abnormalities, and characteristic computed tomography findings. Ten patients (90.9%) were successfully managed conservatively with vitamin K and FFP, while 1 patient (9.1%) underwent diagnostic laparoscopic exploration. The median hospital stay was 6 days. No complications or mortality were observed during treatment. One patient (9.1%) experienced recurrence within 3 months after treatment.

## 6. Limitation

This study has several limitations. Its small sample size and single-center retrospective design limit generalizability, and the absence of a control or comparison group precludes causal inference regarding treatment effectiveness. The 3-month follow-up period was relatively short and did not allow assessment of long-term recurrence or outcomes. Because the analysis was based on retrospective medical records, some clinically relevant data were not uniformly available, including prior bleeding history, longitudinal INR control, post-recovery anticoagulation strategies, and patient-reported perspectives. Formal correlation analysis between initial INR severity and clinical outcomes was not performed because the small sample size would have made such analyses underpowered and potentially misleading. In addition, quantitative radiologic parameters such as bowel wall thickness and intramural attenuation were illustrated in a representative case but were not systematically measured across all patients, limiting imaging-based comparative analysis. Follow-up CT was not routinely performed after clinical improvement, which limited radiologic confirmation of resolution.

## Acknowledgments

The authors would like to thank the staff of Military Hospital 103 for their support in patient care and data collection.

## Author contributions

**Conceptualization:** Khac Thao Thai, Trong Hoe Nguyen, Thanh Son Le, Doanh Hieu Tran.

**Data curation:** Khac Thao Thai, Doanh Hieu Tran.

**Formal analysis:** Khac Thao Thai, Doanh Hieu Tran.

**Investigation:** Khac Thao Thai, Doanh Hieu Tran.

**Methodology:** Khac Thao Thai, Doanh Hieu Tran.

**Project administration:** Doanh Hieu Tran.

**Resources:** Khac Thao Thai, Doanh Hieu Tran.

**Supervision:** Trong Hoe Nguyen, Thanh Son Le.

**Validation:** Khac Thao Thai, Doanh Hieu Tran.

**Visualization:** Khac Thao Thai, Doanh Hieu Tran.

**Writing – original draft:** Khac Thao Thai, Doanh Hieu Tran.

**Writing – review & editing:** Khac Thao Thai, Doanh Hieu Tran.
